# Isolation of mutant alleles of the U6 snRNA m ^6^ A methyltransferase Mtl16 and characterization of their genetic interactions with splicing mutants in *Schizosaccharomyces pombe*

**DOI:** 10.17912/micropub.biology.000948

**Published:** 2023-08-11

**Authors:** Alaina H. Willet, Liping Ren, Lesley A. Turner, Kathleen L. Gould

**Affiliations:** 1 Department of Cell and Developmental Biology, Vanderbilt University School of Medicine, Nashville, TN US

## Abstract

*Schizosaccharomyces pombe* Dim1 is a conserved essential component of the U4/U6.U5 tri-snRNP complex essential for pre-mRNA splicing. In a synthetic lethal screen with the temperature-sensitive *dim1-35* mutant, we isolated multiple alleles of non-essential *mtl16* that encodes the U6 snRNA m ^6^ A methyltransferase. Further genetic analysis revealed strong and specific negative genetic interactions between *mtl16* and a mutation in the Dim1 binding partner, Prp31, and between *dim1-35* and a mutation in the Prp31 binding partner, Prp6. Our work provides additional tools to study pre-mRNA splicing in *S. pombe* and biological confirmation of the importance of the Prp6-Prp31-Dim1-U6 snRNA interactions for pre-mRNA splicing.

**Figure 1. Isolation and characterization of mtl16 mutants:**
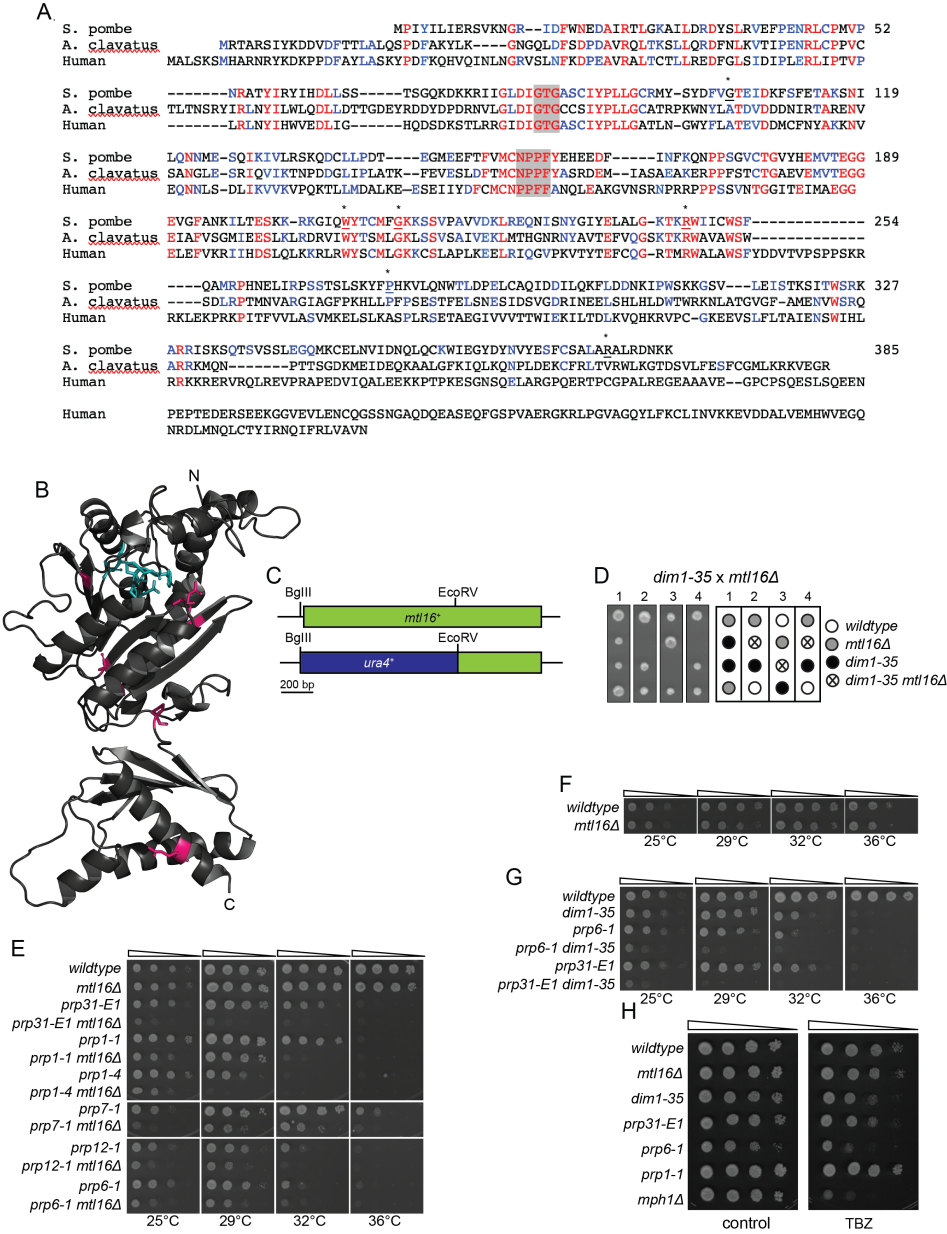
(A) An alignment of *S. pombe* Mtl16 with human and *Aspergillus clavatus* orthologs generated by Clustal Omega (Sievers and Higgins, 2021). Identical amino acids are in red and conserved positions are in blue. Conserved residues essential for m6A methylation (Ruszkowska et al., 2018) are shaded in grey. The amino acids of the *S. pombe* Mtl16 sequence are indicated at the right. The positions of amino acids mutated in the seven *mtl16* mutant strains (two were identical) are indicated with asterisks and underlining. (B) Structural model of *S. pombe* Mtl16 using AlphaFold2 (Jumper et al., 2021; Varadi et al., 2022) with positions of mutations synthetically lethal with *dim1-35* indicated in magenta. Amino acids necessary for catalytic activity (Ruszkowska et al., 2018) shaded in A are highlighted in cyan. (C) Schematic of the *mtl16* deletion construct. The open reading frame is shown in green and the *ura4+* sequences inserted between the BglII and EcoRV sites are in blue. Because the BglII site is 20 base pairs upstream of the first ATG codon, the disruption construct resulted in a null allele. (D) The *mtl16* deletion is synthetically lethal with *dim1-35* . Four tetrads on YE plates incubated at 25˚C from the indicated cross and a schematic of relevant gentoypes are shown. (E-H) The indicated strains were grown in liquid YE (E, G and H) or EMM (F) at 25˚C until they reached mid-log phase. Then, 10-fold serial dilutions were made and 2.5 µL of each was spotted on YE agar plates and incubated at the indicated temperatures for 3-5 days prior to imaging. Thiabendazole (TBZ) at 17.5 µg/mL was added to the YE plates in H.

## Description

Description Dim1 is a highly conserved and essential thioredoxin-like protein that is part of the U4/U6.U5 tri-snRNP splicing complex (Berry and Gould, 1997; Gottschalk et al., 1999; Reuter et al., 1999; Stevens and Abelson, 1999; Zhang et al., 2000; Zhang et al., 1999) . The *S. cerevisiae* ortholog is termed Dib1 (first named CDH1) (Berry and Gould, 1997) . The *S. pombe dim1-35* mutant, in which amino acid 126 is changed from G to D, is defective in pre-mRNA splicing (Carnahan et al., 2005) , displays significant cell cycle defects (Berry and Gould, 1997) , and is synthetically lethal with a mutant in the anaphase-promoting complex, *lid1-6* (Berry et al., 1999) . To identify other Dim1 interactors, we characterized additional mutants obtained in the screen in which *lid1-6* was isolated (Berry et al., 1999) . Briefly, a *dim1-35* strain with mouse *dim1* cDNA expressed from the *nmt1* promoter inserted at the *leu1* locus was mutagenized with nitrosoguanidine as described (Moreno et al., 1991) . Colonies that were inviable in the presence of thiamine were isolated as reported (Berry et al., 1999) . Seven of the isolated strains that were backcrossed three times and then outcrossed were found to contain second-site mutations unlinked to the *dim1* locus. The new alleles were not temperature-sensitive on their own. Further genetic analyses indicated that all 7 of these strains contained mutations in the same gene and they were not *lid1* alleles. A genomic library constructed in the pUR18 vector (Barbet et al., 1992) was transformed into one of the 7 mutant strains using standard procedures (Moreno et al., 1991) and colonies that survived in the presence of thiamine were isolated and the rescuing plasmids recovered. DNA sequencing revealed that all of the recovered plasmids contained the non-essential *mtl16+* gene (Ishigami et al., 2021; Lock et al., 2018) . Mtl16 is a 44 kDa highly conserved methyltransferase responsible for m6A modification of the U6 small nuclear (sn) RNA in fission yeast and human (Pendleton et al., 2017) ( Figure 1A ); this enzyme is not present in *S. cerevisiae* . While the human ortholog, METTL16, is essential, *S. pombe* Mtl16 facilitates pre-mRNA splicing of just a subset of transcripts via m6A methylation of U6 snRNA and its deletion displays only a mild pre-mRNA splicing defect (Ishigami et al., 2021; Pendleton et al., 2017) . The *mtl16* gene was amplified from each of the 7 isolated strains and sequenced to determine the positions of the inactivating mutations ( Figure 1A and B ). Two alleles were identical, changing amino acid 104 from G to D due to a single G to A nucleotide change. Two other strains had single base mutations that resulted in G215D and R247C substitutions. In two other strains at positions W209 and R378, respectively, single base changes caused the introduction of premature stop codons. The final *mtl16* mutation was caused by a frame shift that truncated the Mtl16 protein at P276 and added non-sensical amino acids to its tail ( Figure 1A and B ). That a small C-terminal truncation disrupted *mtl16* function is concordant with the high confidence prediction of an alpha helix in that region of the protein ( Figure 1B ). The *mtl16* gene was disrupted by replacing sequences from the BglII site to the indicated EcoRV site in the coding region with *ura4+* , creating a null allele by removing the start codon ( Figure 1C ). *mtl16::ura4+* was synthetically lethal with *dim1-35* ( Figure 1D ) but viable on its own at a range of temperatures on both YE media, as described previously (Ishigami et al., 2021; Lock et al., 2018) and EMM ( Figure 1E and 1F). This is consistent with the mild splicing defects previously noted for *mtl16∆* cells (Ishigami et al., 2021) . To gain insight into the specificity of the strong negative interaction with *dim1-35* , we constructed a suite of double mutant strains containing *mtl16::ura4+* and other splicing mutants. We found that *mtl16::ura4+* showed a strong negative genetic interaction with *prp31-E1* (Bishop et al., 2000) but had milder negative interactions with temperature-sensitive alleles of *prp1, prp7, prp12* and *prp6* ( Figure 1E ). Like *dim1* , *prp31* encodes a component of the U4/U6.U5 tri-snRNP and *prp31-E1* is synthetically lethal with *prp6-1* (Bishop et al., 2000) . *dim1-35* also showed strong negative genetic interactions with *prp31-E1* and *prp6-1* ( Figure 1G ). Many mutations in pre-mRNA splicing factors display cell cycle phenotypes, particularly defects in mitotic progression that are primarily due to faulty pre-mRNA splicing (Burns and Gould, 1999; Burns et al., 2002; Kallgren et al., 2014; Somma et al., 2020; Valcarcel and Malumbres, 2014) . The *dim1-35* and *mtl16∆* strains are both reported to have sensitivity to the microtubule destabilizing drug, thiabendazole (TBZ), suggesting an impact on transcripts necessary for spindle function (Berry and Gould, 1997; Ishigami et al., 2021) . In the case of *dim1-35* , this is consistent with the significant mitotic defects of *dim1-35* cells (Berry and Gould, 1997) . To determine if mutations in interacting genes share this same sensitivity, we tested the TBZ sensitivity of *prp31-E1, prp6-1* , and *prp1-1* using the mitotic checkpoint defective mutant, *mph1∆* (He et al., 1998) , as a control. While *prp31-E1* and particularly *prp6-1* were sensitive to TBZ, unexpectedly, we did not detect sensitivity of *mtl16∆* to TBZ. Thus, TBZ sensitivity appears to be a common phenotype associated with defective pre-mRNA splicing and perhaps related to the level of splicing deficiency. Structures of the U4/U6.U5 tri-snRNP determined by cryo-electron microscopy indicate that Dib1/Dim1 is situated adjacent to the invariant ACAGAGA sequence of U6 snRNA in the catalytic core of the spliceosome (Nguyen et al., 2016; Wan et al., 2016) . It is the third base in this invariant sequence, residue 37 in the *S. pombe* U6 snRNA, that is modified by Mtl16 (Gu et al., 1996) . These structures also suggest that Dib1/Dim1 binds directly to Prp31, and that Prp31 binds Prp6 through a distinct interface (Nguyen et al., 2016; Wan et al., 2016) . Thus, our genetic interaction data provide biological support for the importance of these predicted physical interactions, indicate that Dim1, Prp31 and Prp6 cooperate with U6 snRNA to promote spliceosome activation, and provide additional tools with which to investigate the role of U6 snRNA pre-mRNA splicing in S. pombe.

## Methods

Yeast methods: *S. pombe* strains were grown in yeast extract (YE) or Edinburgh minimal medium (EMM) supple­mented with appropriate amino acids with appropriate supplements and standard *S. pombe* mating, sporulation, and tetrad dissection techniques were used for backcrossing, outcrossing, and to construct new strains (Moreno et al., 1991) . EMM with 5 µg/ml thiamine was used to repress expression of murine *dim1* from the *nmt1* promoter. To disrupt the *mtl16* gene, a portion of the protein coding region in a pUC18 genomic clone was replaced with *ura4+* ( Figure 1C ) and the resultant construct was transformed into KGY246 and Ura4 ^+^ colonies were selected. These were grown for multiple generations and a stable Ura4 ^+^ integrant was selected. Disruption of the *mtl16* gene was confirmed by colony PCR.

Molecular biology methods : Plasmids were constructed using standard molecular biology techniques. *mtl16* alleles were sequenced by generating a PCR product with an oligonucleotide 100 bp upstream of the start site (CAACCGCCGATAAAGGCGATATAG) and 100 bp downstream of the stop codon (GGCTATTCAATATAAGAAGATTACCAA) (Integrated DNA technologies). The PCR product was sequenced with two additional oligonucleotides; a forward oligonucleotide at 635 bp within the mtl16 genomic DNA (CTTACTCCCTGATACCGAAGGGATG) and a reverse oligonucleotide at 780 bp within the genomic mtl16 DNA (CATTTCATGATAAACTCCAGTACAAACAC).

## Reagents

The strains used in this study and their genotypes are listed below.

**Strain Genotype Source**

KGY246 *ura4-D18 leu1-32 ade6-M210 h ^-^* Lab stock

KGY1193 *mtl16-R247C leu-32 ura4-D18* *h ^-^* This study

KGY1194 *mtl16-G104D leu-32 ura4-D18* *h ^-^* This study

KGY1195 *mtl16-W209stop leu-32 ura4-D18* *h ^-^* This study

KGY1196 *mtl16-R378stop leu-32 ura4-D18* *h ^-^* This study

KGY1197 *mtl16-P276stop leu-32 ura4-D18* *h ^-^* This study

KGY1198 *mtl16-G104D leu-32 ura4-D18* *h ^-^* This study

KGY1201 *mtl16-G215D leu-32 ura4-D18* *h ^-^* This study

KGY1224 *mtl16-G104D dim1-35 leu1-32::nmt81-dim1* This study

*ade6-M216* h-

KGY1301 *mtl16::ura4 ^+^ ura4-D18 leu1-32 ade6-M21X h ^-^* This study

KGY1844 *mtl16::ura4 ^+^ ura4-D18 leu1-32 ade6-M21X h ^+^* This study

KGY2457 *prp31-E1 ade6-M210* *leu-32 ura4-D18* *h ^-^* (Bishop et al., 2000)

KGY1142 *prp1-1 ura4-D18 leu1-32 ade6-M21X h ^-^* (Potashkin et al., 1989)

KGY8109 *prp1-1 mtl16::ura4 ^+^ ura4-D18 leu1-32* This Study

*ade6-M21X h ^-^*

KGY2551 *prp1-4 ura4-D18 leu1-32 ade6-M210 h ^-^* (Urushiyama et al., 1996)

KGY8110 *prp1-4 mtl16::ura4 ^+^ ura4-D18 leu1-32* This Study

*ade6-M210 h ^-^*

KGY7218 *prp7-1 ura4-D18 leu1-32 ade6-M210 h ^-^* (Potashkin et al., 1998)

KGY8468 *prp7-1 mtl16::ura4 ^+^ ura4-D18 leu1-32* This Study

*ade6-M21X h ^+^*

KGY8432 *prp12-1 ura4-D18 leu1-32 ade6-M210 h ^-^* (Urushiyama et al., 1996)

KGY8543 *prp12-1 mtl16::ura4 ^+^ ura4-D18 leu1-32* This Study

*ade6-M21X h ^-^*

KGY1877 *prp6-1 ura4-D18 leu1-32 ade6-M210 h ^-^* (Potashkin et al., 1998)

KGY8544 *prp6-1 mtl16::ura4 ^+^ ura4-D18 leu1-32* This Study

*ade6-M21X h ^+^*

KGY2671 *dim1-35 leu1-32:nmt81-dim1cDNA* *ura4-D18 h ^-^* Lab stock

KGY8111 *mtl16::ura4 ^+^* *prp31-E1 ura4-D18 h ^+^* This study
